# A transcriptomic-guided strategy used in identification of a wheat rust pathogen target and modification of the target enhanced host resistance to rust pathogens

**DOI:** 10.3389/fpls.2022.962973

**Published:** 2022-09-02

**Authors:** Bernard Nyamesorto, Hongtao Zhang, Matthew Rouse, Meinan Wang, Xianming Chen, Li Huang

**Affiliations:** ^1^Department of Plant Sciences and Plant Pathology, Montana State University, Bozeman, MT, United States; ^2^USDA-ARS, Cereal Disease Laboratory, Department of Plant Pathology, University of Minnesota, Saint Paul, MN, United States; ^3^Department of Plant Pathology, Washington State University, Pullman, WA, United States; ^4^Wheat Health, Genetics, and Quality Research Unit, United State Department of Agriculture-Agriculture Research Service, Pullman, WA, United States

**Keywords:** *MYC4* Transcription factor, *Triticum aestivum* (bread wheat), rust, (*Puccinia*), BSMV-VIGS, kallisto program, wheat-tilling, enhanced resistance

## Abstract

Transcriptional reprogramming is an essential feature of plant immunity and is governed by transcription factors (TFs) and co-regulatory proteins associated with discrete transcriptional complexes. On the other hand, effector proteins from pathogens have been shown to hijack these vast repertoires of plant TFs. Our current knowledge of host genes' role (including TFs) involved in pathogen colonization is based on research employing model plants such as *Arabidopsis* and rice with minimal efforts in wheat rust interactions. In this study, we begun the research by identifying wheat genes that benefit rust pathogens during infection and editing those genes to provide wheat with passive resistance to rust. We identified the wheat *MYC4* transcription factor (TF) located on chromosome 1B (*TaMYC4-1B)* as a rust pathogen target. The gene was upregulated only in susceptible lines in the presence of the pathogens. Down-regulation of *TaMYC4-1B* using barley stripe mosaic virus-induced gene silencing (BSMV-VIGS) in the susceptible cultivar Chinese Spring enhanced its resistance to the stem rust pathogen. Knockout of the *TaMYC4-1BL* in Cadenza rendered new resistance to races of stem, leaf, and stripe rust pathogens. We developed new germplasm in wheat via modifications of the wheat *TaMYC4*−*1BL* transcription factor.

## Introduction

Plants employ a complex network of signaling pathways to defend themselves against pathogen attacks. Signal integration is dictated by transcription factor (TF) regulatory networks. Transcriptional reprogramming is a major component of plant immunity and is administered by TFs and co-regulatory proteins connected within distinct transcriptional complexes (Moore et al., 2011). Over a couple of decades, studies have uncovered numerous TF family members mostly in *Arabidopsis thaliana* and rice that are critical in regulating proper defense responses when plants are confronted by pathogens. Many of these TFs have been categorized into AP2/ERF, bHLH, bZIP, MYB, NAC and WRKY families. *MYC*, a basic-helix-loop-helix (bHLH) family TFs were initially discovered from a homology study between an oncogene carried by the Avian *v*irus, *My*elo*c*ytomatosis (*v-MYC*) and a human gene overexpressed in different cancers, *c*ellular *MYC* (*c-MYC*). They have a DNA binding domain made of 50–60 amino acids, which allows for homo- or heterodimerization to their DNA consensus hexamer sequence CANNTG (Finver et al., 1988). The bHLH TFs have been shown to be key regulators in Jasmonic Acid (JA)-mediated defense responses and in mediating crosstalk with other phytohormones, including salicylic acid (SA), abscisic acid (ABA), gibberellins (GA), and auxin (Kazan and Manners, 2013).

Co-evolved with host defense systems in plants, pathogens are also continually developing counter mechanisms to overcome host defenses. It has become evident that one component of their arsenal is manipulating host cellular processes using effector proteins, including exploiting pathogen susceptible host genes. Efforts in modifying these pathogen targeted host genes to increase resistance against pathogens have become a go-to approach for disease-resistant breeding in model crops such as rice. For example, alteration of rice promoters *OsSWEET14* and *OsSWEET11* and the *OsMPK5* gene enhanced resistance to bacterial blight (caused by *Xanthomonas oryzae* pv. *oryzae*), bacterial panicle blight (*Burkholderia glumae*) and blast (caused by fungus *Magnaporthe grisea*) of rice (Li et al., 2012; Jiang et al., 2013; Xie and Yang, 2013). Similarly, alteration of wheat *TaMLO* and *TaEDR1* genes (three homeologs) enhanced resistance to powdery mildew (caused by *Blumeria graminis* f. sp. *tritici*) (Shan et al., 2013; Wang et al., 2014; Zhang et al., 2017). Identifying host targets of pathogen effectors and modifying these sites to abolish the effector-target interactions would be a quick approach to generate new resistance.

Upon the release of the complete genome sequence of hexaploid wheat (*Triticum aestivum* L.) by the International Wheat Genome Sequencing Consortium (Appels et al., 2018), it has become apparent that of the over 107,891 high-confidence genes identified, more than 35,000 are transcriptional factors categorized into 40 families and 84 subfamilies (Appels et al., 2018). This genome resource makes gene identification in wheat from a short sequence read possible. In a pilot transcriptomic study on the interaction between wheat and the leaf rust pathogen (*Puccinia triticina; Pt*) using a pair of near isogenic lines (NILs) of mnr220/MNR220 (the Alpowa background) (Campbell et al., 2012), the expression patterns of some wheat genes were found to be upregulated starting at 5-days post-*Pt* inoculation (data not shown), a critical rust *Pt* development stage when the rust has established infection sites in susceptible wheat cultivars. Among those upregulated expressed genes, five of them are transcriptional factors (TFs). Among the five TFs, one of them is 97% similar to a cDNA of *TaMYC4*-like sequence named TRIUR3_32014 from *Triticum urartu* (a close wild relative of wheat) after BLAST search in NCBI. MYC2/MYC3/MYC4 were identified as a core of TFs regulating jasmonic acid (JA) and JA-isoleucine accumulation through a positive amplification loop in Arabidopsis (Van Moerkercke et al., 2019). This observation suggested a hypothesis that some rust pathogens upregulate host JA production to suppress SA-mediated defense by manipulating host MYC transcription factors. The wheat *MYC4* genes (*TaMYC4*) are the targets of the rust pathogen during infection. In this study, we have demonstrated that the *TaMYC4* homeolog in the long arm of chromosome 1B (*TaMYC4*−*1BL*) is upregulated upon pathogen infection and that modification of the *TaMYC4*−*1BL* enhanced resistance to wheat against rust pathogens.

## Materials and methods

### Plant and pathogen materials

Spring wheat cultivar Alpowa (PI 566596) was obtained from the USDA National Plant Germplasm System (NPGS), and Chinese Spring (CS) was obtained from Dr. Evans Lagudah at Commonwealth Scientific and Industrial Research Organization (CSIRO). Cadenza, spring wheat, was obtained from the SeedStor *via* http://www.seedstor.ac.uk.

The *Puccinia graminis* f. sp. *tritici* (*Pgt*) races QFCSC (isolate 10UML6-1) and TPMKC (isolate 07MT137-2) were provided by Dr. Yue Jin from Cereal Disease Laboratory, USDA-ARS, St. Paul, MN. The *P. triticina* (*Pt*) race PBJJG (isolate 09KSAL1-6) was provided by Dr. Robert Bowden (USDA-ARS, Manhattan, KS, USA). A *P. striiformis* f. sp. *tritici* (*Pst*) culture (race and isolate unknown) was collected from the Bozeman Agricultural Research and Teaching Farm of Montana State University (MSU).

### Plant growth conditions

For rust screenings, wheat seeds were directly planted into 4-inch small pots (5 seeds/pot) containing SunGro Horticulture Sunshine mix (HeavyGardens Company, Denver, CO). For seed propagation and crosses, wheat seeds were first germinated in Petri dishes on filter paper at room temperature. At root radical emergence, the seeds were transferred to 8-inch pots (one seedling/pot) containing a 1:1 ratio mixture of local soil: Sunshine mix. Growth conditions were set at 22/14°C Day/night temperatures and a 16 h photoperiod in a greenhouse at the Plant Growth Center, MSU. Plants were watered and fertilized every day with Peters General Purpose Plant Food (Scotts-Miracle-Gro Company, Marysville, OH, USA) at a concentration of 150 ppm.

### Rust pathogen inoculation and assessment

All rust screenings were completed at the two-leaf seedling stage. Rust inoculations were conducted as described in Campbell et al. (2012). In brief, plants were inoculated with rust pathogen urediniospores in Soltrol170 oil suspensions. Inoculated plants were then transferred to a Percival I-60D dew chamber (Percival Scientific Inc., Perry, IA, USA) pre-conditioned to an internal air temperature between 15 and 17°C for *Pt* and *Pst*, 19-20°C for *Pgt* for 24 h. An additional step of 4 h exposure to light prior to removing the plants from a dew chamber for stem rust inoculation. *Pgt* races QFCSC and TMLKC, *Pt* race PBJJG and *Pst* evaluations were conducted at Montana State University in the greenhouse of the Plant Growth Center. Assays with TTKSK (isolate 04KEN156/04) and TKTTF (isolate 13GER10-5) were completed in the Cereal Disease Laboratory, USDA-ARS, St. Paul, MN, following the procedure described by Jin et al. (2007). The tests with other *Pst* races were conducted at the Wheat Health, Genetics, and Quality Research Unit, USDA-ARS, Pullman, WA, according to the procedure described by Line and Qayoum (1992) and infections types (IT) recorded 18–20 days after inoculation.

Infection types of seedlings to leaf and stem rusts were assessed using the 0-4 IT scale (McIntosh et al., 1995) at 8 and 13–14 days post inoculation (dpi) for *Pt* and *Pgt*, respectively, when the symptoms and signs of susceptible controls were fully expressed. Stripe rust was assessed at 14 dpi based 0 (immune)—9 scale (highest susceptible) (Line and Qayoum, 1992).

### Sample collection and treatments

For RNA extraction, leaf samples were taken from three plants separately per treatment. Each sample was frozen in liquid nitrogen and stored at −80°C. RNA extractions were completed when all the samples at different time points were collected. Sample collection times ranged from 0 to 10 dpi, depending on the experiment. The samples at 0 dpi were taken immediately after inoculation before placing the inoculated plants in a dew chamber. For DNA extraction, leaf samples were taken and immediately used for extraction.

### RNA and DNA extraction

Total RNAs were isolated and treated with DNase I on columns using the Qiagen RNeasy Plant Mini Kit (Qiagen, Valencia, CA) following the manufacturer's instructions. Genomic DNA extraction for conventional PCR was done using the QIAGEN DNeasy Plant Mini Kit (Qiagen Sciences Inc, Germantown, MD, USA). For KASP assays, DNA was extracted from 96 plants using the 96-well plate extraction procedure modified from Holleley and Sutcliffe (2022). The quality and concentration of total RNA/DNA were assessed using 260/280ABS measurements on a NanoDrop 1,000 spectrophotometer (Thermo Fisher Scientific Inc., Wilmington, DE, USA). The integrity of DNA or RNA was checked *via* agarose gel electrophoresis with 2 μl of a sample, 4 μl of water, 1 μl loading buffer (98% formamide, 10 mM EDTA, 0.25% bromophenol blue, and 0.25% xylene cyanol) on a 0.8–1% gel stained by GelRed (Bio-Rad, Hercules, CA) at 125 volts for 25 min.

### qRT-PCR, conventional PCR and KASP assays

qRT-PCR was performed using the iScript One-Step RT-PCR Kit with SYBR Green (Bio-Rad, Hercules, CA) on a CFX96 real-time PCR detection system (Bio-Rad, Hercules, CA) following the manufacturer's procedure with 100–150 μg sample RNA at annealing temperatures of 56/57°C depending on the primers. ACTB (β-actin) (Kozera and Rapacz, 2013) was used as the housekeeping gene for normalization of transcript abundance (Supplementary Table 1). qRT-PCR was conducted in triplicate.

PCR amplifications were conducted in 20 μl reactions containing 25 mM MgCl_2_, 10 mM dNTP, 2 μM of each primer (BN1BL primers, **Table 2**), 50 ng genomic DNA and 1 unit Go Taq Flexi DNA polymerase (Promega, Madison, Wisconsin). Amplifications were performed at 95°C for 7 min, followed by 35 cycles at 95°C for 45 s, 55°C for 45 s, and 68/72°C for 40 s (depending on primers), with a final extension at 68/72°C for 10 min.

KASP genotyping was conducted using the KASP genotyping trial kit following the manufacturer's protocol (Biosearch Technologies Genomic analysis by LGC) using manually designed KASP primers (Supplementary Table 1) on a CFX96 real-time PCR detection system. Products from qRT-PCR, PCR and KASP were checked using gel electrophoresis as described previously.

### Barley stripe mosaic virus-induced gene silencing assay

Gene knockdown was conducted *via* a BSMV-VIGS assay. The original BSMV vectors were obtained from Dr. Andrew O. Jackson (UC Berkeley, CA, USA). The target fragment for the silencing assay was inserted into the modified γ vector ready for direct PCR cloning as described by Campbell and Huang (2010). BSMV RNA transcripts were synthesized *in vitro* using the T7 RNA polymerase (New England Biolabs, Ipswich, MA, USA) from linearized α, β, and γ plasmids. The BSMV inoculum was prepared with 3 μL of BSMV RNAs (1:1:1 ratio of α, β, and γ) and 22.5 μL of the inoculation buffer. The inoculum was then rub-inoculated onto the first leaf of two-leaf-stage plants. Leaf tissue was sampled 9 days after virus inoculation to test the silencing efficiency. Stem rust inoculations were done 14 days post virus inoculation when BSMV-induced target gene silencing reached the highest level.

### Mutant search and validation

Cadenza mutants were identified from the wheat-tilling database using the sequence of candidate genes as a query. Wheat-tilling is a resource TILLING population consisting of 2,700 individuals developed *via* ethyl methanesulfonate (EMS) mutagenesis in tetraploid durum cv “Kronos” and the hexaploid wheat cv “Cadenza” backgrounds (Rakszegi et al., 2010). The genome of each mutant has been completely sequenced. Mutations of requested mutants were validated *via* sequencing of the target regions after PCR amplification from the wild-type Cadenza and mutants using gene-specific primers.

### Genetic analysis

Genetic analysis was conducted to test the genotype-phenotype association using 150 seeds from a self-pollinated heterozygote mutant plant at the selected locus. Also, 96 F_4_ individuals were used for genotyping and phenotyping *via* KASP assay using the designed KASP primers. For mutant L683F, the single nucleotide polymorphism (SNP) was a C-T nucleotide change from the wild-type to the L683F mutant. The forward oligos were designed as: Allele 1 with wild-type nucleotide (C) and Allele 2, which has the mutant nucleotide (T). A common reverse primer was designed for both allele oligos. A combination of the three oligos was used in the assay (Supplementary Table 1).

### Pathogenesis-related genes expression

During the time courses of the *Pgt* TMLKC infections, *PR* gene expression was assayed at 0, 1, and 2 dpi in both the wild-type Cadenza and mutant. Leaf samples were collected from three biological replicates per dpi for the wild-type and mutant and pretreated under recommended conditions for RNA extraction. Using the corresponding *PR* gene primers (Supplementary Table 1), *PR* genes were quantified using extracted RNA *via* real-time-qPCR as described earlier. The PR gene primers were according to Desmond et al. (2005).

### Databases and *in silico* sequence analysis

All BLAST and sequence downloads were completed using the International Wheat Genome Sequence Consortium (IWGSC) resources at https://wheat-urgi.versailles.inra.fr/Seq-Repository/BLAST. Initially, sequence downloads were completed from IWGSC RefSeq v1.0 (2017-08-07) and later from IWGSC RefSeq v2.1 (2019-02-20). Multiple sequence alignments were conducted using ClustalW Omega at https://www.ebi.ac.uk/Tools/msa/clustalo/. Gene and conserved domain predictions were performed using Softberry at http://www.softberry.com/berry.phtml?topic=fgenesh&group=programs&subgroup=gfind and Pfam at http://pfam.xfam.org/. Primers were designed either manually or using the PrimerQuest^®^ tool at https://www.idtdna.com/pages/tools/primerquest, and primer specificity was assessed by BLAST search of the IWGSC database. RNA-seq data quality was checked using FastQC Version 0.11.6 (Babraham Bioinformatics, Cambridge, CB22 3AT, UK).

The RNA sequence data from the Sequence Read Archive (SRA) of the National Center for Biotechnology Information (NCBI) accession number (PRJEB12497: http://www.ebi.ac.uk/ena/data/view/PRJEB12497) (Dobon et al., 2016) were used to quantify the expression levels of homeologs at different time points *via* Kallisto software (Bray et al., 2016). The wheat transcriptome downloaded from IWGSC was indexed and reads of RNA-seq were also assessed for quality *via* FastQC High Throughput Sequence QC Report (version: 0.11.7) in interactive mode. Paired-end sequences were split using Fastq-dump prior to transcript quantification. The paired-end reads were run using default parameters with 100 bootstraps (-b 100). Refer to Kallisto Manual-Pachter Lab (https://pachterlab.github.io/kallisto/manual) for more details to run Kallisto on bulk RNA-seq data.

### Statistical analysis

Data assessment and analysis were conducted in Microsoft Excel and R-studio software version 1.1.453.0. For real-time PCR assays, data were used only if the Ct standard deviation among the triplets was ≤ 0.2 and the mean of the triplet's Ct was used for downstream analysis. Relative expression was calculated using the ΔΔCt method as described in the CFX96 manual (Bio-Rad, Hercules, CA), where fold change = 2^−ΔΔCt^. Expression measurement of genes was conducted in three technical replicates for each of three biological replicates. Standard deviations were calculated among three biological replicates or using pooled standard deviation formula when comparing two different groups (ΔCt values). Student's *t*-tests were performed to test whether the expression levels at different time points were significantly different. The *p*-values were calculated based on an unpaired two-tailed distribution. Expression patterns were graphically represented using averages of the three biological replicates.

## Results

### Conserved domains of *TaMYC4* genes in wheat

To address our question of *TaMYC4*'s possible negative role in wheat plant defense against rust pathogens, we began with further investigation of *TaMYC4* conserved domains to relate its structure to function. We identified three copies of the *TaMYC4* gene in bread wheat using the cDNA of *TaMYC4*-like sequence of TRIUR3_32014 from *T. urartu* to BLAST search the International Wheat Genomic Sequence Consortium (IWGSC) database. *TaMYC4*-like genes were identified on 1AL, 1BL, and 1DL chromosomes with 97–99% similarities on the DNA level or the amino acid level with an Expect Value of 0.0. The three homeologs were predicted to have conserved basic-helix-loop-helix (bHLH) and helix-loop-helix (HLH) domains at E-values approximately 0.0. A Leucine-zipper domain was predicted on only the 1BL homeolog at an E-value of 0.31. Each of these domains is a classical *MYC4* conserved domain (Supplementary Figure 1, Supplementary File 2). The bHLH/HLH and leucine zipper motifs allow binding of MYC proteins to DNA and dimerization with other bHLH TFs (including Max). Based on a ClustalW Omega multiple sequence alignment of the sequences, the percent identity matrix indicated that the three homeologs had over 95% identity at the DNA, mRNA, and protein levels. These insights gave additional validity that the sequence we were interested in is a TF of *MYC4*.

### Expression profiles of the *TaMYC4* genes by qRT-PCR

**Figure 1:**
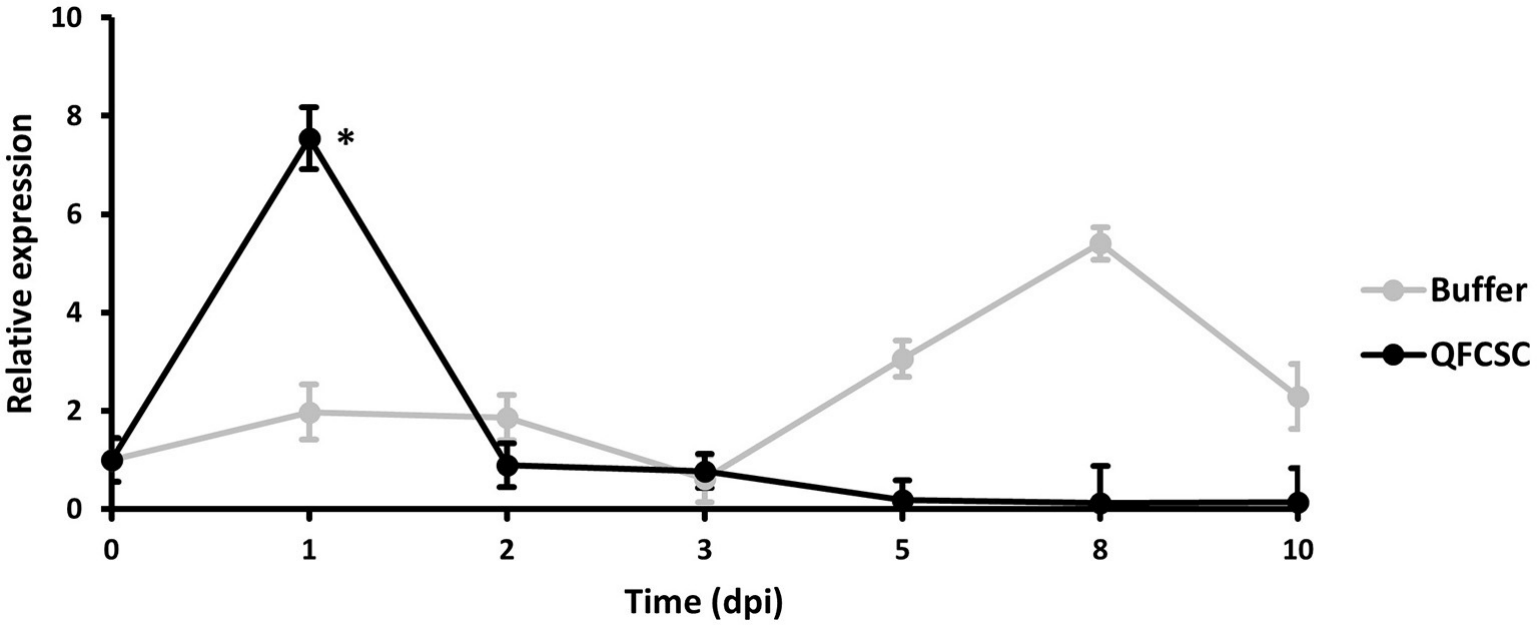
Relative expression of *TaMYC4* in wheat cultivar Alpowa inoculated with buffer (control) and the *Puccinia graminis* f. sp. *tritici* (*Pgt*) pathogen (race QFCSC). Alpowa cultivar were inoculated with *Pgt* urediniospore at 2-leaf stage. RNA samples were extracted from the leaf samples collected at seven time points. Real-time PCR was used to quantify transcript abundances of *TaMYC4* genes in both buffer and *Pgt* inoculated samples. Expression of *TaMYC4* genes at each time point was computed relative to the level at 0-dpi. Error bars represent standard deviation computed as the square root of pooled variance between groups and * denote statistical significance at the *P* ≤ 0.05 levels calculated between each time point compared with 0 dpi.

To further confirm the expression pattern of the *TaMYC4* genes observed in the pilot study (data not shown), we conducted a time-course study in Alpowa, the genetic background of the NILs used in the wheat-*Pt* transcriptomics pilot study. Alpowa was inoculated with *Pt* race PBJJG and *Pgt* race QFCSC or buffer Soltrol 170 isoparaffin as a mock inoculated control. An RNA collection time course was set at 0-, 1-, 2-, 3-, 4-, 5-, 8-, and 10-days post-inoculation (dpi). The expression level of *TaMYC4* was assayed *via* qRT-PCR using the BN4RT primers that measured all three homeologs of the *TaMYC4* genes (Supplementary Table 1, Supplementary File 1). We did not observe any significant changes in the expression profile of *TaMYC4* at any of the time points for the PBJJG-inoculated Alpowa (Supplementary Figure 2). When Alpowa was inoculated with the *Pgt* race QFCSC, the expression levels of *TaMYC4* were similar in samples taken at 0 dpi between *Pgt*-inoculated Alpowa and the control. However, a significant increase in *TaMYC4* transcript abundance was detected at 1 dpi in *Pgt* infected Alpowa compared to the control plants. The *TaMYC4* expression level returned to an undetectable level at 2 dpi and stayed unchanged during the rest of the time points (Figure 1). The expression level of *TaMYC4* in buffer inoculated Alpowa showed minor and insignificant changes along from 1 to 3 dpi but showed an increase from 5 to 8 dpi that then declined at 10 dpi. This increase was, however, statistically insignificant. A significantly higher expression of *TaMYC4* occurred after rust infection and only in the susceptible host revealed in the pilot study. These observations encouraged us to investigate whether *Pgt*-induced *TaMYC4* upregulation is beneficial to the pathogen during colonization of wheat.

### Silencing of the *TaMYC4* genes

**Figure 2:**
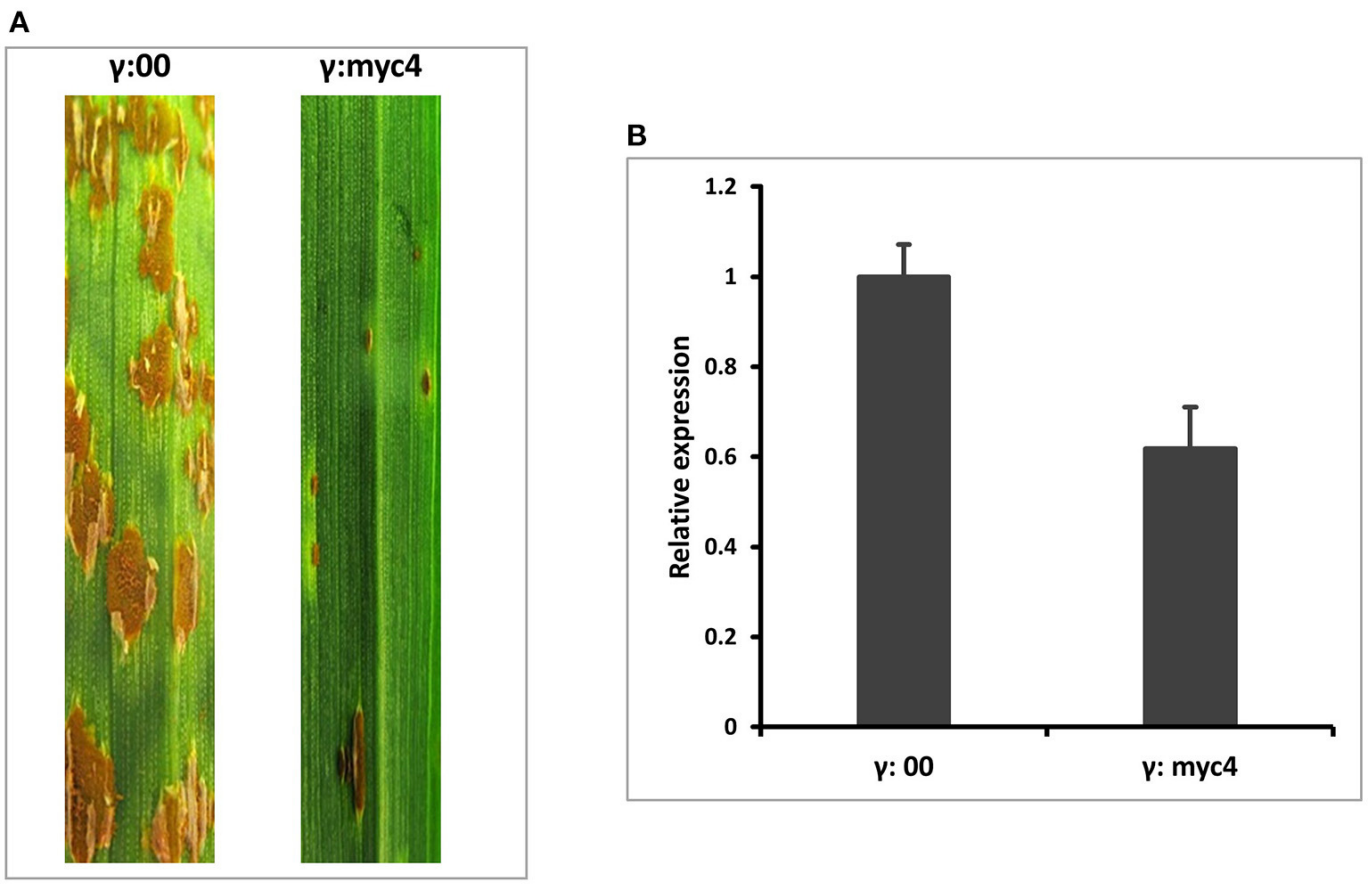
BSMV-VIGS of *TaMYC4* in wheat cultivar Chinese Spring (CS). The first leaf of each plant was rub inoculated with the indicated BSMV constructs at the two-leaf stage and then spray inoculated with *Pgt* race QFCSC at 10 days after BSMV inoculation. The disease was assessed and photographed at 14 dpi. CS without any viral inoculation, used as a rust inoculation control; γ:00, CS inoculated with BSMV: *MYC4*; γ:*myc4*. RNA was extracted from viral-free leaf samples taken from γ:00 and γ:*myc4* plants prior to pathogen inoculation. Transcript abundance was quantified via RT-qPCR. Error bars represent standard deviation among three biological reps. **(A)** Infection types of *MYC4*-silenced plants **(B)** Relative expression of *TaMYC4* in γ:00 and γ:*myc4* silenced plants.

To quickly determine the function of *TaMYC4* regarding stem rust resistance, we downregulated the *TaMYC4* genes and examined the infection types on the host. We knocked down all three endogenous copies of *TaMYC4* (to avoid functional redundancy) in a rust susceptible wheat cultivar Chinese Spring (CS) using a BSMV-VIGS assay. We used CS instead of Alpowa for the silencing assay for two reasons. One is that BSMV-VIGS is homolog-based, and the sequences of the three *TaMYC4* genes obtained from the IWGSC database are from CS. The other reason was to validate our hypothesis in a different cultivar background to study if the pathogen uses the same strategy to infect different cultivars. A construct containing a 247-bp fragment conserved among the gene homeologs after multiple sequence alignment (Supplementary File 1) was obtained *via* PCR amplification using primers VIGS-F/R (Supplementary Table 1) and used to silence each homoeolog on the three chromosomes (labeled as BSMV:*MYC4*) (see also Supplementary File 1). A construct carrying only the BSMV genome was used as a no-target control and labeled as BSMV:00. For short, the BSMV-derived construct with no insert was named as γ00, and each BSMV silencing construct was named after the target gene, for example, γ*MYC4*. The concurrent silencing of BSMV inoculum was made by combining the α: β: γ target transcripts in an equal ratio with excess inoculation buffer (FES). In each assay, 20 wheat seedlings were inoculated with γ*MYC4* or γ00, as a control. At 6 days post BSMV inoculations (dpbi), viral symptoms were visualized on the newly emerged leaves of plants inoculated with BSMV. At nine dpbi, plants inoculated with BSMV constructs showed viral-symptom-free leaf segments, indicating that BSMV induced gene silencing had occurred. Three viral-symptom-free leaf segments were randomly sampled from plants inoculated with γ00 and γ*MYC4* construct for RT-qPCR. The plants were inoculated with *Pgt* race QFCSC immediately. Infection type (IT) observed 14 dpi showed enhanced disease resistance in plants that had *TaMYC4* silenced. Non-silenced plants were susceptible (Figure 2A. Transcript abundances of *TaMYC4* were measured through qRT-PCR using primers BN4RTF/R (Supplementary Table 1), which confirmed a 40% reduction of *TaMYC4* in silenced plants relative to the control (Figure 2B). Though the reduction in the expression of the three *TaMYC4* homeologous genes resulted in enhanced resistance to *Pgt* QFCSC, we do not know which homeolog or if all three of them are critical for the rust fungus to colonize the wheat host successfully.

### Expression of *TaMYC4* homeologs during the *Pst* infection

We explored whether all three *TaMYC4* homeologs are negatively involved in the plant's defense mechanism based on their expressions. We quantified each *TaMYC4* homeolog *via* Kallisto software using the RNA sequence data generated from a previous study (Dobon et al., 2016) involving Avocet *Yr5* (resistant) and Vuka (susceptible) inoculated with *Pst* pathogen at 0, 1, 2, 3, and 5 dpi and accessed from the NCBI. We used just the *Pst*-wheat data set since we could not find readily available *Pgt*-wheat RNAseq data for the specific time-course study of our interest. Also, the *Pst*-wheat study helped us to assess the *TaMYC4* expression pattern in the wheat-*Pst* interaction, which we already examined beforehand in *Pgt/Pt*-wheat (Supplementary Figures 2; Figure 1). FastQC is a quality control tool for high throughput sequence data. Overall, FastQC checks on the RNA-seq data indicated good quality features such as per base sequence quality and overrepresented sequences. The RNA-seq was pseudo-aligned and quantified *via* Kallisto software (Bray et al., 2016). The output of transcript abundance was recorded in transcript per million (tpm). The transcript abundances of the genes of interest (1AL, 1BL, and 1DL) were imported using the gene IDs; TRIAE_CS42_1AL_TGACv1_000298_AA0008240.1, TRIAE_CS42_1BL_TGACv1_726352_AA2170300.1, TRIAE_CS42_1DL_TGACv1_061684_AA0201690.1, respectively. Pre-examination of the transcript data for abundance satisfied normality requirement in the R studio. A graph of expression of the gene was plotted using the averages of biological replicates at each time point.

**Figure 3:**
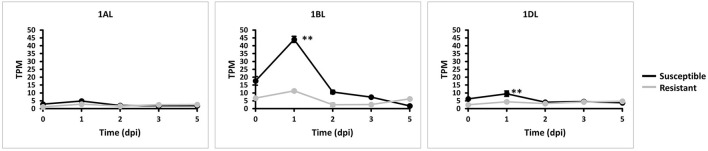
Expression of *TaMYC4* (1AL, 1BL and 1DL) homeologs during stripe rust–Avocet Yr5 (resistant) and Vuka (susceptible) interactions. TPM is transcript per million. Error bars represent standard deviation among three biological reps and ** denote statistical significance at the *P* ≤ 0.01 levels calculated between each time point and 0 dpi.

Homeolog *TaMYC4-1BL* was significantly upregulated in the susceptible cultivar at 1 dpi compared with the resistant cultivar. It was also the most expressed of the three homeologs (Figure 3). This result led us to the search for permanent alteration (mutants) of *TaMYC4-1BL*.

### Identification of *TaMYC4* mutants and their response to the rust pathogens

Using the *TaMYC4-1BL* cDNA sequence as a query, a BLAST search for matches in the database of wheat-tilling mutant lines revealed more than 40 lines carrying a mutation on the *TaMYC4-1BL* gene in the wheat cv Cadenza background. We selected three mutants that had a Sorting Intolerant from Tolerant (SIFT) score of 0.0. Two of the three mutations were confirmed after genotyping with *TaMYC4*−*1BL* specific primers (Supplementary Table 1). One is a homozygote with a missense mutation at the protein position 683, changing amino acid L to F, hereafter identified as L683F-MYC4−1BL or L683F. The other is a heterozygote with a missense mutation from M to I at position 635, designated as M635I-MYC4-1BL or M635I (Table 1).

**Table 1:** Summary of identified TaMYC4−1BL Cadenza mutants.

**Chromosome**	**Nucleotide** **change**	**Mutated amino** **acid position**	**Amino acid** **changes**	**Type of** **mutation**	**SIFT** **score^a^**	**Mutated** **ID^b^**
1BL	G to A	683	L to F	Homozygote	0	L683F-MYC4−1BL
1BL	C to T	635	M to I	Heterozygote	0	M635I-MYC4−1BL

a SIFT score predicts whether an amino acid substitution affects protein function, and ranges from 0 to 1. The amino acid substitution is predicted to be damaging if the score is ≤ 0.05 and tolerated if the score is >0.05.

b Gene IDs were based on amino acid change and position of change, gene name and chromosome.

**Figure 4:**
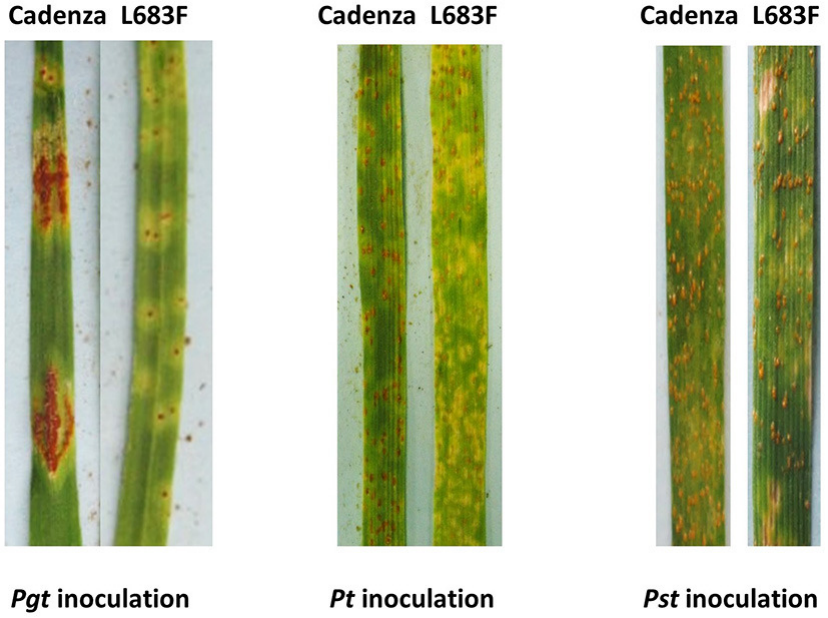
Reactions of the wild-type Cadenza and the *TaMYC4* mutant L683F after inoculation with *Puccinia graminis* f. sp. *tritici* (*Pgt*), *P. triticina* (*Pt*) and *P. striiformis* f. sp. *tritici* (*Pst*) inoculation. Infection types (IT) of Cadenza: L683F in each pair of images were, *Pgt* (4: 1+), *Pt* (3: 2-;) and *Pst* (4: 3c) Photos of *Pgt* and *Pst* inoculated plants were taken 14 days post inoculation. Photos of *Pt* inoculated plants were taken 9 days post inoculation.

The Cadenza L683F mutant showed enhanced resistance to *Pgt* race TPMKC, *Pt* race PBJJG, and one field-collected race of *Pst* (Figure 4; Table 2). The L683F mutant was then tested using additional races of *Pgt* and *Pst* (Table 2). The mutant showed higher levels of resistance to *Pgt* QFCSC and *Pst* race PSTv-4. However, the mutant was as susceptible as Cadenza to *Pgt* races TTKSK and TRTTF, and *Pst* races PSTv-37, PSTv-41 and PSTv-47. The rust screening results indicated the resistance conferred by the mutation L683F in *TaMYC4-1B* is race-specific.

**Table 2:** Rust evaluations of Cadenza L683F mutant and wild-type Cadenza.

	**Infection type (IT)**					
**Pathogen**	**Race**	**Isolate**	**Alpowa**	**Avocet**	**Mutant**	**Cadenza**
*Pgt*	QFCSC	10UML6-1	3+	N	1	2
	TPMKC	07MT137-2	3+	N	;1-	3+
	TTKSK	04KEN156/04	N	N	3+	2+3
	TRTTF	06YEM34-1	N	N	3+	3+
*Pt*	PBJJG	09KSAL1-6	N	N	;1	3
*Pst*	NK	18Field Collection	N	N	3	7
	PSTv-4	19WA-200-YrSP	N	8	3	3
	PSTv-37	19ID-11	N	8	8	8
	PSTv-41	19WA-193	N	8	8	8
	PSTv-47	19ID-32	N	8	8	8

Pgt, P. graminis f. sp. tritici; Pt, P. triticina; Pst, P. striiformis f. sp. tritici. For infection type, higher the number, more susceptible plant with a “+” (more than average) or a “–” (less than average) to further quantify the level. A semicolon (;) symbolizes the presence of hypersensitive flecks. N means not tested. Wheat cultivar Alpowa was used as a stem rust susceptible control, and Avocet was used as stripe rust susceptible control. The race of the field collected stripe rust used is unknown (NK).

### Genetic analysis of the mutations

To confirm the new rust resistance in the mutant due to the mutation in *TaMYC4-1B* and not due to other mutations in the background, we crossed the mutant L683F with a susceptible cultivar Alpowa. F_1_ plants were self-pollinated to produce F_2_ segregating populations. Out of the 150 individuals in the first genetics analysis, infection types of 148 plants with clear phenotypes were scored. A resistant to susceptible ratio of 35:113 was observed, fitting the 1:3 ratio, [χ^2^ (1, *N* = 148) = 0.14, *p* = 0.70], indicating that resistance was a recessive phenotype. The genotyping results from 12 susceptible and 3 resistant plants confirmed the resistant plants had the mutant nucleotide (T/A) and the susceptible plants had the (C/G) base. The second step of confirmation was to design a KASP assay to detect the SNP for mutant L683F using the design primers (Supplementary Table 1) based on the *TaMYC4-1BL* scaffold sequence (Supplementary File 1). The result of the KASP assay from 94 F_2_ individuals and the two parental lines showed that all 35 resistant plants had a monomorphic SNP marker of L683F. Among the 58 susceptible plants, 17 were heterozygotes of the SNP, and 42 were homozygotes of the wild-type SNP.

### Molecular mechanism of the new rust resistance

**Figure 5:**
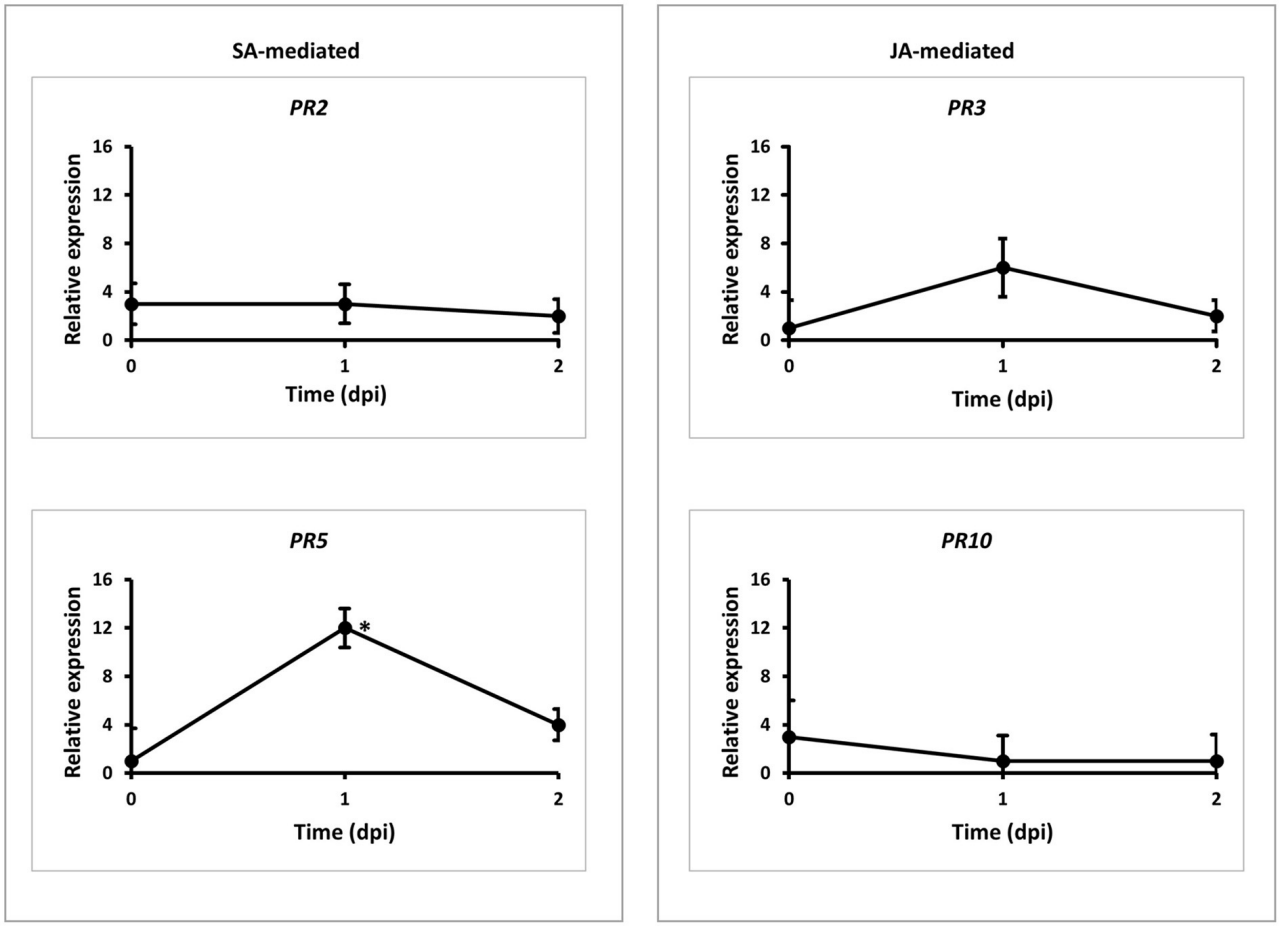
Expressions of pathogen related (PR) genes responding to *Pgt* (race TMLKC) in the wild-type and mutant wheat lines. Wild-type Cadenza and mutant *L683F-MYC4-1BL* were inoculated with the *Pgt* pathogen at 2-leaf stage. RNA samples were extracted from the leaf samples collected at three time points. Real-time PCR was used to quantify transcript abundances of the four *PR* genes. Expression of PR genes at each time point was expressed in the mutant relative to the level in the wild-type. * denote statistical significance at the *P* ≤ 0.05 levels compared between wildtype and mutant at each time point. Error bars represent standard deviation computedas the square root of pooled variance between groups.

To understand the genetic mechanism of the new rust resistance, we tested the expressions of four *PR* genes, including SA-dependent *PR2* and *PR5* and JA-dependent *PR3* and *PR10* (Van Loon and Van Strien, 1999) in a time-course study in the wild-type Cadenza and mutant L683F with *Pgt* TPMKC infection. The level of each *PR* gene was monitored at three-time points with qRT-PCR using *PR* gene-specific primers (Supplementary Table 1). The basal expression of the *PR* genes at 0 dpi was at a similar level between the wild-type Cadenza and mutant L683F (Figure 5), about 0-0.5 relative to the expression of the reference gene *actin* (data not shown). The result suggested that these four *PR* genes had minimal expression in the absence of *Pgt*. However, *PR5* was highly upregulated (12-fold) at 1 dpi in L683F relative to its expression in the wild-type Cadenza (Figure 5), suggesting that the mutation in L683F permitted an elevated level of an SA-mediated *PR* gene. Meanwhile, *PR2, PR3*, and *PR10* had no significant differences compared to the wild-type Cadenza across the three-time points. This result suggested that the mutation in L683F did not affect the two JA-mediated *PR* genes in the defense response to *Pgt* TPMKC infection.

## Discussion

### Wheat *TaMYC4* gene aids rust pathogens in host colonization

The first evidence that supports the hypothesis that the *TaMYC4* genes are targets of the pathogen for assistance in colonization is the upregulation of *TaMYC4* only in rust inoculated but not in the mock-inoculated wheat plants. This provided initial evidence that differentially expressed *TaMYC4* TF was due to the presence of rust pathogens (Figure 1). Secondly, the upregulation of *TaMYC4*-*1B* occurred only in a susceptible host (Figure 3). The expression trajectory indicated that the targeting of the *TaMYC4-1B* gene happened at the early stage of pathogen colonization. Also, down-regulation of all three copies of *TaMYC4* reduced the susceptibility of a susceptible host to *Pgt* race QFCSC (Figure 2A). The relative reduction in *TaMYC4* transcript abundance in the silenced plants (Figure 2B) indicated that the observed enhanced resistance was attributable to the *TaMYC4* knockdown. The silencing assay suggested that the effect of the *TaMYC4* upregulation was a benefit to the pathogen and negative to the wheat host. *Arabidopsis MYC4* has been shown positively involved in JA and JA-isoleucine accumulation (Van Moerkercke et al., 2019). JA and ABA are positive regulators of stomatal closure (Sarwat and Tuteja, 2017). Bacterial pathogen *Pseudomonas syringae* secret coronatine (COR), a structural and functional analog of the active form of JA, to open host stomata during infection (Zhou et al., 2015). We hypothesize that rust pathogens upregulate *TaMYC4-1B* to increase the JA level to open up host stomata because rust fungal germ tubes enter the host through the stomata. Additional support of this hypothesis is a study of *PR* gene expression during rust pathogen infections. JA-mediated *PR* gene expressions were low in resistant lines, suggesting a low JA level at the early time point post-inoculation (Zhang et al., 2018). Finally, a recessive loss-of-function mutation in *TaMYC4*−*1BL* conferred resistance to rust pathogens (Figure 4), supporting our claim that the *TaMYC4-1B* gene facilitated infection by rust pathogens in a compatible wheat host.

The three homeologs of *TaMYC4* have bHLH/HLH conserved domains characteristic of *MYC* transcription factors. Nonetheless, an LZ domain is only found in the *TaMYC4-1B* protein (Supplementary Figure 1), suggesting a possible non-redundancy function under different conditions. Most literature have provided evidence of the positive contribution of the leucine zipper TFs to biotic (Milligan et al., 1998; Ballvora et al., 2002; Alves et al., 2013) and abiotic stresses (Yu et al., 2020). Generally, LZ TFs heterodimerize with other proteins involved in cell proliferation, survival, and metabolism (Adhikary and Eilers, 2005). These functions could be hijacked by pathogens for their benefit with enhanced host susceptibility, as observed with the mutation of *TaMYC4-1B* resulting in enhanced resistance to the rust pathogens. More research is however needed to arrive at this conclusion. Indeed, several studies (Shitsukawa et al., 2007; Hovav et al., 2008; Chaudhary et al., 2009; Chen et al., 2011) have revealed transcriptional divergence among homeologs. However, non-redundancy can also arise due to different expression patterns, not due to the protein sequence. Therefore, we suggest that the *TaMYC4*−*1BL* gene acts as a rust pathogen susceptibility gene such that it acts as a factor needed by the pathogens to colonize the host.

The segregation ratio of resistant to susceptible phenotypes confirmed that *TaMYC4*−*1BL* confers a recessive phenotype for resistance or a dominant phenotype for susceptibility. At 24 h post-inoculation, development of haustorial mother cells of a wheat rust fungus commences (Serfling et al., 2016). Haustoria are known to play a vital role in cellular communication between pathogen and host (Heath, 1997), nutrient acquisition (Hahn and Mendgen, 2001), manipulation of host metabolism, and the suppression of host defenses (Voegele and Mendgen, 2003). This knowledge further strengthens our claim that *TaMYC4-1BL* was a target to manipulate host defense to benefit the pathogen at the early pathogenesis stage.

Indeed, the higher level of *TaMYC4*−*1BL* transcripts at 1-day post *Pgt* inoculation (Figure 1) resulted in lower *PR5* expression in Cadenza L683F mutant (Figure 5), suggesting that *PR5* expression was hampered when the TF was increased in the presence of *Pgt*. *PR* genes are involved in host defense under different wheat-pathogen race interactions (Zhang et al., 2018). *PR5* is an SA-dependent thaumatin-like protein (Van Loon, 1982) and has been shown to inhibit the growth of various fungi (Muthukrishnan et al., 2001). Strategies of bacteria hijacking plant hormones to manipulate host defense has been well studied. For example, *Agrobacterium tumefaciens* uses its T-DNA to facilitate production of host auxin and cytokinin hormones in the formation of crown galls. Various strains of *P. syringae* produces coronatine (COR) phytotoxin to manipulate host hormones to enhance bacterial growth and symptom development (Mittal and Davis, 1995). It was later shown that COR is structurally similar to JA isoleucine hence functioning in antipathy to the SA pathway, which plays a crucial role in defense against this bacterial (Brooks et al., 2005; Browse, 2009). Zheng et al. (2012) demonstrated that COR targets host NAC TFs to case stomata reopening and systemically induced susceptibility. We suspect that *PR5* might have been suppressed by rust pathogen effectors using *TaMYC4*−*1BL* as a host target gene. The insignificant expression of *PR10* (the other SA-related proteins) in this interaction (Figure 5) suggested that for specific signal transduction, separate *PR* proteins may be involved during different plant-pathogen interactions. Also, it has been established that a cross-communication between SA- and JA-dependent defense pathways exists (Felton and Korth, 2000; Pieterse et al., 2001). The relatively lower and insignificant expression levels of the JA-pathway associated *PR* proteins (*PR2* and *PR3*) at 1 dpi (Figure 5) allude to this crosstalk that enables plants to fine-tune their defense reactions depending on the type of stress they encounter.

The bHLH superfamily of transcription factors, including *TaMYC4*, have essential regulatory components in transcriptional networks of many developmental pathways (Atchley and Fitch, 1997). In *Arabidopsis, MYC4* TFs are known to bind to the G-box of promoters and are involved in JA gene regulation (Niu et al., 2011). Collectively, *MYC4, MYC2* and *MYC3* were shown to control JA-dependent responses (Fernández-Calvo et al., 2011). It was demonstrated that *MYC4* could form complexes with glucosinolate-related MYBs to regulate glucosinolate biosynthesis (Schweizer et al., 2013). A recent study unraveled that an *MYC2*/*MYC3*/*MYC4*-controlled positive-feedback loop transcriptionally regulated spray-induced jasmonate accumulation (Van Moerkercke et al., 2019). These activities of *MYC4* in *Arabidopsis* give additional credence to our hypothesis that *TaMYC4* is beneficial to pathogens in suppressing host defense in the early stages of wheat rust interaction. However, contrary to its implication in JA-pathways in *Arabidopsis*, we found its negative function in the SA-dependent pathway implicated in *PR5* suppression. This emphasizes our knowledge that a gene could function in different pathways in different species and under varying conditions. At this point, this study has not ascertained if this mechanism is similar for all the three rust pathogens and has also not uncovered the detailed mode of action of *TaMYC4-1BL* during the compatible wheat-rust interactions. Hence further study is necessary to elucidate these unknowns.

### Cadenza L683F mutant showed race-specific resistance

The Cadenza L683F mutant showed resistance to *Pt* race PBJJG, *Pgt* race TPMKC, but not to *Pgt* races TTKSK (Ug99) and TRTTF. Also, the mutant was susceptible to all the *Pst* races tested except for a field-collected uncharacterized *Pst* isolate and race PSTv-4 (Table 2). The race-specific resistance was shown by hypersensitive reaction and moderate resistance at the seedling stage during their interaction with the pathogens. The resulting infection types range from 1 to 3 in seedlings. The wild-type Cadenza had a moderate resistance level to *Pgt* race QFCSC, but the mutant L683F had enhanced resistance to the pathogen. Furthermore, the same mutation enhanced resistance to three rusts, suggesting a common target in a host used by the rust pathogens as a strategy.

We also noticed that down-regulation of the *TaMYC4* genes in wheat cv. CS enhanced resistance to *Pgt* race TPMKC, and mutated *TaMYC4-1BL* in different wheat cultivars could enhance resistance to the same rust pathogen race, suggesting a rust pathogen race used the same strategy targeting the same genes in different genetic backgrounds of the same host.

### An approach to creating new resistant germplasm

Over the years, wheat rust resistance breeding has been focused on using adult-plant resistance (APR) and all-stage resistance (R) genes from wheat and related species. At the same time, this is very resource-consuming because these resistance genes are continually overcome by evolving virulent races of rust pathogens, mainly because most of these genes confer race-specific resistance. There have been considerations for the building resistance gene cassettes to confer efficient resistances against different rust pathogens or races. However, this effort requires the availability of effective resistance genes as resources.

Due to the limitations of natural existing resistance genes in wheat or its relatives, scientists are looking for strategies to generate new resistance genes *via* mutagenesis or genome editing. Decades of studies on plant-pathogen interactions revealed that when pathogens successfully colonize host plants, some of the host genes are reprogrammed by the pathogens. Those host genes could be transcriptionally activated by the pathogen transcription activator-like effectors (TALEs), interact with the pathogen effectors to suppress the plant defense response (Fukuoka et al., 2009), or redirect the host nutrient sinks to the host-pathogen interfaces (Chen et al., 2010). In nature, a single mutation of one of those host genes could happen and make the host less desirable to the pathogen, therefore less severity of the disease. For example, rice *xa13*-mediated resistance is due to a mutation of the effector binding site (EBE) in the promoter of the host glucose transporter gene OsSWEET11 (*Xa13*). The mutation leads to the loss of the bacterial pathogen *Xanthomonas oryzae pathovar oryzae* (Xoo) strain PXO99 *PthXo1*-mediated induction of the *SWEET* gene and reduced bacterial growth (Chen et al., 2010). In wheat, changes of only two amino acids in a conserved region of a hexose transporter at the *Lr67* locus between *lr67* and *Lr67* reduce the growth of multiple biotrophic pathogen species (Moore et al., 2015). Understanding the specificity of pathogen targeting DNA sequences has enabled the development of new host resistance in rice (Hummel et al., 2012; Zeng et al., 2015), pepper (Romer et al., 2009), and resistance against *Ralstonia solanacearum* in a broad host range (de Lange et al., 2013).

Our study demonstrated an effective scheme of using a transcriptomic-guided approach in wheat to select candidates of pathogen targets, a BSMV-induced gene silencing assay to identify and confirm the targets, and phenotype and genetic analysis of mutations on selected targets to develop new resistant germplasm. *TaMYC4*-1BL was selected because the upregulation of the gene was only detected in susceptible lines in the presence of rust pathogens. A reverse genetic approach using BSMV-induced gene silencing revealed that the host demonstrated improved resistance when the *TaMYC4-1BL* was downregulated. With the known DNA sequence of *TaMYC4-1BL* and an EMS mutagenized population, we were able to identify mutations in the gene. Phenotype and genetic analysis of the L683F mutation in *TaMYC4-1BL* of Cadenza confirmed a new resistance to rust pathogens in wheat. This approach provides a means of navigating the challenges associated with germplasm creation and studying gene function in wheat, low efficiency in wheat transformation, and concerns with detrimental effects of a host gene mutation.

## Data availability statement

The datasets presented in this study can be found in online repositories. The names of the repository/repositories and accession number(s) can be found in the article/Supplementary material.

## Author contributions

Conceptualization and funding acquisition: LH. Methodology: BN, LH, HZ, MR, MW, and XC. Investigation and formal analysis: BN. Writing—original draft: BN and LH. Writing—review and editing: BN, LH, MR, MW, and XC. All authors contributed to the article and approved the submitted version.

## Funding

This research was supported by the NSF BREAD program (Grant No. IOS-0965429).

## Conflict of interest

The authors declare that the research was conducted in the absence of any commercial or financial relationships that could be construed as a potential conflict of interest.

## Publisher's note

All claims expressed in this article are solely those of the authors and do not necessarily represent those of their affiliated organizations, or those of the publisher, the editors and the reviewers. Any product that may be evaluated in this article, or claim that may be made by its manufacturer, is not guaranteed or endorsed by the publisher.
