# Supplementary material for: A transcriptomic-guided strategy used in identification of a wheat rust pathogen target and modification of the target enhanced host resistance to rust pathogens
Source: Front Plant Sci. 2022 Sep 2;13:962973. doi: 10.3389/fpls.2022.962973 (PMC9478542; doi:10.3389/fpls.2022.962973)
Supplement: Supplementary file 1 [file Data_Sheet_1.docx]

Supplementary Material

**Figure S1**. Predicted conserved domains of *MYC4* homeologs. Gene and conserved domains were predicted using mRNA and protein sequences of wheat *MYC4* gene copies (1AL, 1BL and 1DL) from IWGSC via Softberry.com and Pfam respectively. bHLH= basic helix-loop-helix; HLH= helix-loop-helix; LZ= Leucine-zipper. Figure is not drawn to scale.

**
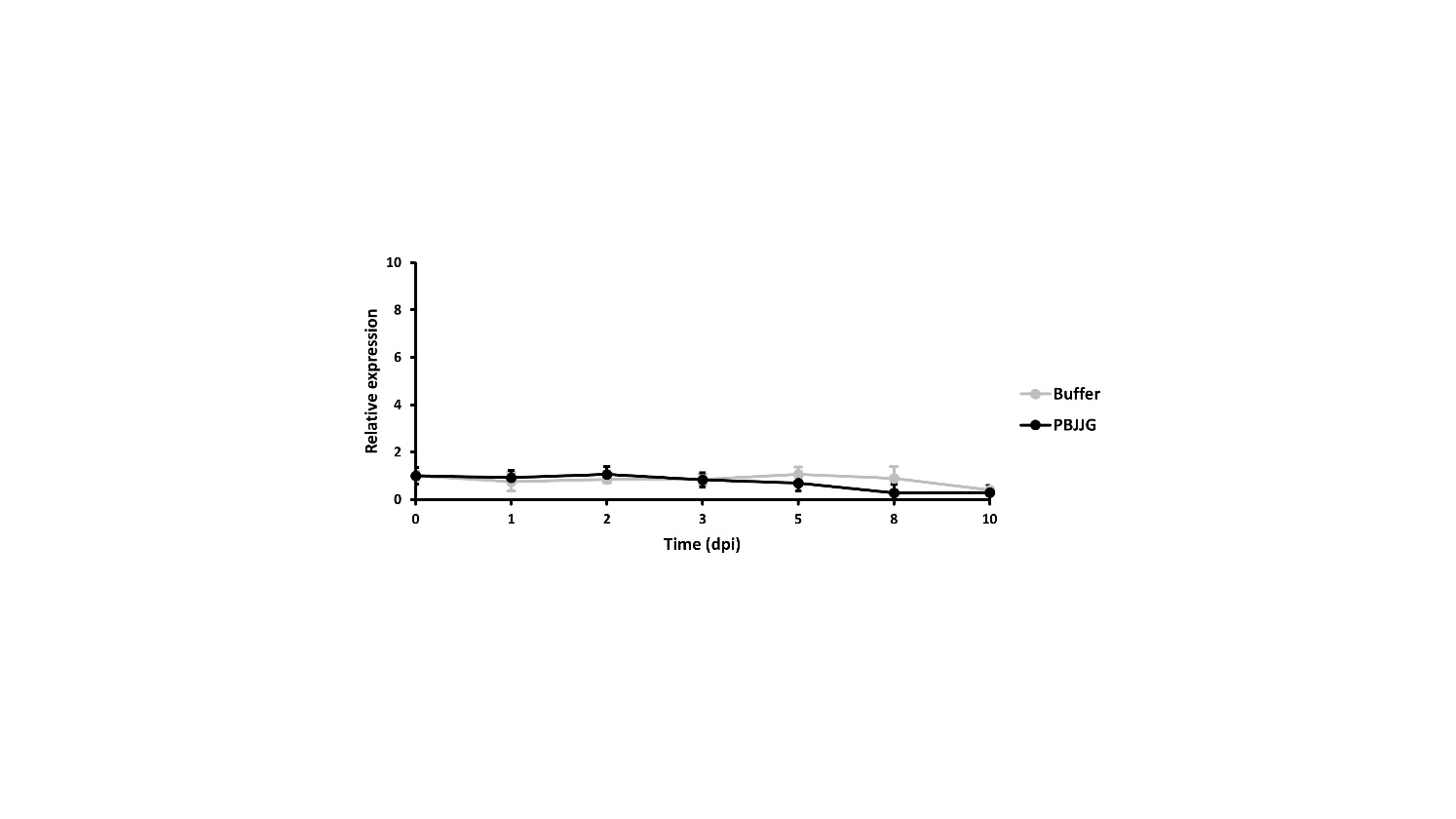
**

**Figure S2.** Relative expression of *TaMYC4* in wheat cultivar Alpowa inoculated with buffer (control) and *Pt* (race PBJJG). Alpowa cultivar were inoculated with *Pt* urediniospore at 2-leaf stage. RNA samples were extracted from the leaf samples collected at seven time points. Real-time PCR was used to quantify transcript abundances of *TaMYC4* genes in both buffer and *Pt* inoculated samples. Expression of *TaMYC4* genes at each time point was computed relative to the level at 0-dpi. Error bars represent standard deviation computed as the square root of pooled variance between groups and * denote statistical significance at the P ≤ 0.05 levels calculated between each time point compared with 0 dpi.

# Table S1: Summary of primers

| Primer name | Purpose | Tm (°C) | Sequence 5’ – 3’ |
| --- | --- | --- | --- |
| VIGS-F/R | Gene knockdown |  | F: GACGCTCCATTCCCAAATCG  R: TCATGATGTCCTTCACCACG |
| BN4RT | qRT-PCR | 56 | F: TCCGCACCATGGTCTGCAT  R: TCCGCCGTTGAGGTTGAAGAG |
| BN1BL | PCR | 55 | F: ATGATCCGCGTGCAGTG  R: GAGTAATAGGATCGCGGTTTCC |
| ACTIN | qRT-PCR | 57 | F: CCAGCAATGTATGTCGCAATCC  R: CCAGCAAGGTCCAAACGAAGG |
| PR2 | qRT-PCR | 57 | F: CTCGACATCGGTAACGACCAG  R: GCGGCGATGTACTTGATGTTC |
| PR3 | qRT-PCR | 57 | F: AGAGATAAGCAAGGCCACGTC  R: GGTTGCTCACCAGGTCCTTC |
| PR5 | qRT-PCR | 57 | F: ACAGCTACGCCAAGGACGAC  R: CGCGTCCTAATCTAAGGGCAG |
| PR10 | qRT-PCR | 57 | F: TTAAACCAGCACGAGAAACAT  R: ATCCTCCCTCGATTATTCTCACG |
| Allele1 | KASP assay | 70 | GAAGTTGACCAAGTTCATGCTTGCAGTG  CCACAAGCGCAACCAC**C** |
| Allele2 | KASP assay | 70 | GAAGGTCGGAGTCAACGGATTTGCAGTG  CCACAAGCGCAACCAC**T** |
| CP † | KASP assay | 70 | CCCGGGTGGCCATCTTCACCGCCACC |

† CP= common primer

**File S1**: **Alignment of *TaMYC4* homeologs. VIGS insert is highlighted green.**

_C_T3= cDNA of *TaMYC4* -like sequence named TRIUR3_32014 from *Triticum urartu*

cT3 ------------------------------------------------------------ 0

1BL ATGAACCTGTGGACGGACGACAACGCCTCCATGATGGAGGCCTTCATGGCCTCCGCCGAC 60

1AL ------------------------------------------------------------ 0

1DL ATGAACCTGTGGACGGACGACAACGCCTCCATGATGGAGGCCTTCATGGCCTCCGCCGAC 60

cT3 ------------------------------------------------------------ 0

1BL ATGCCGGCCTTCCCATGGGGCGCGGCGGCCACCCCGCCGCCGCCGGCCGCCGTCCCGCAG 120

1AL ------------------------------------------------------------ 0

1DL ATGCCGGCCTTCCCCTGGGGCGCTGCGGCCACCCCGCCGCCGCCGGCCGCCGTGCCGCAG 120

cT3 ------------------------------------------------------------ 0

1BL CAGCCGGCCTTCAACCAGGACACGCTGCAGCAACGCCTGCAGGCCATCATCGAGGGCTCC 180

1AL ---------------------------------CGCCTGCAGGCCATCATCGAGGGCTCC 27

1DL CAGCCGGCCTTCAACCAGGACACGCTGCAGCAGCGCCTGCAGGCCATCATCGAGGGCTCC 180

cT3 ------------------------------------------------------------ 0

1BL AGGGAGACCTGGACCTACGCCATCTTCTGGCAGTCCTCCACCGACGCCGGCGCCTCGCTC 240

1AL AGGGAGACCTGGACCTACGCCATCTTCTGGCAGTCCTCCACCGACGCCGGCGCCTCGCTC 87

1DL AGGGAGACCTGGACCTACGCCATCTTCTGGCAGTCCTCCACCGACGCCGGCGCCTCGCTC 240

cT3 ------------------------------------------------------------ 0

1BL CTCGGCTGGGGCGACGGCTACTACAAGGGCTGCGACGACGCCGACAAGCGCCGCCAGCAG 300

1AL CTCGGCTGGGGCGACGGCTACTACAAGGGCTGCGACGACGCCGACAAGCGCCGCCAGCAG 147

1DL CTCGGCTGGGGCGACGGCTACTACAAGGGCTGCGACGACGCCGACAAGCGCCGCCAGCAG 300

cT3 ------------------------------------------------------------ 0

1BL CCCACCCCGGCCTCCGCCGCCGAGCAGGAGCACCGCAAGCGCGTCCTCAGGGAGCTCAAC 360

1AL CCCACCCCGGCCTCCGCCGCCGAGCAGGAGCACCGCAAGCGCGTCCTCCGGGAGCTCAAC 207

1DL CCCACCCCGGCCTCCGCCGCCGAGCAGGAGCACCGCAAGCGCGTCCTCCGGGAGCTCAAC 360

cT3 ------------------------------------------------------------ 0

1BL TCGCTCATAGCCGGGGGAGGCGCCGCTGCGCCCGACGAGGCCGTCGAGGAGGAGGTCACG 420

1AL TCGCTCATAGCCGGGGGAGGCGCCGCCGCGCCCGACGAGGCCGTCGAGGAGGAGGTCACG 267

1DL TCGCTCATAGCCGGGGGAGGCGCCGCCGCGCCCGACGAGGCCGTCGAGGAGGAGGTCACG 420

cT3 ------------------------------------------------------------ 0

1BL GACACCGAGTGGTTCTTCCTCGTCTCCATGACCCAGTCCTTCCCCAACGGGATGGGCTTG 480

1AL GACACCGAGTGGTTCTTCCTCGTCTCCATGACCCAGTCCTTCCCCAACGGGATGGGCTTG 327

1DL GACACCGAGTGGTTCTTCCTCGTCTCCATGACCCAGTCCTTCCCCAACGGGATGGGCTTG 480

cT3 ------------ATGGCCATGGTGGTTGATGGTGCAGGCCCCGCGGGGCTCGCCAGCGCG 48

1BL CCGGGGCAGGCGCTCTTCGCCGGCCAGCCCACCTGGATCGCCACGGGGCTCGCCAGCGCG 540

1AL CCGGGCCAGGCGCTCTTCGCCGGCCAGCCCACCTGGATCGCCACGGGGCTCGCCAGCGCG 387

1DL CCGGGCCAGGCGCTCTTCGCCGGCCAGGCCACCTGGATCGCCACGGGGCTCGCCAGCGCG 540

* * * * ** *****************

cT3 CCCTGCGAGCGGGCCAGGCAGGCCTACACCTTCGGCCTCCGCACCATGGTCTGCATCCCC 108

1BL CCCTGCGAGCGGGCCAGGCAGGCCTACACCTTCGGCCTCCGCACCATGGTCTGCATCCCC 600

1AL CCCTGCGAGCGGGCCAGGCAGGCCTACACCTTCGGCCTCCGCACCATGGTCTGCATCCCC 447

1DL CCCTGCGAGCGGGCCAGGCAGGCCTACACCTTCGGCC**TCCGCACCATGGTCTGCAT**CCCC 600

************************************************************

**BN4RT-F**

cT3 CTCGGCACCGGCGTGCTCGAGCTCGGCGCCACCGAGGTCATCTTCCAGACCAACGATAGC 168

1BL CTCGGCACCGGCGTGCTCGAGCTCGGCGCCACCGAGGTCATCTTCCAGACCAACGATAGC 660

1AL CTCGGCACCGGCGTGCTCGAGCTCGGCGCCACCGAGGTCATCTTCCAGACCAACGATAGC 507

1DL CTCGGCACCGGCGTGCTCGAGCTCGGCGCCACCGAGGTCATCTTCCAGACCAACGATAGC 660

************************************************************

cT3 TTGGGGAGGATCCGCTCGCTCTTCAACCTCAACGGCGGAGGAGGGGGCTCTGGATCCTGG 228

1BL TTGGGGAGGATCCGCTCGCTCTTCAACCTCAACGGCGGAGGAGGGGGCTCTGGATCCTGG 720

1AL TTGGGGAGGATCCGCTCGCTCTTCAACCTCAACGGCGGAGGAGGGGGCTCTGGATCCTGG 567

1DL TTGGGGAGGATCCGCTCG**CTCTTCAACCTCAACGGCGGA**GGAGGGGGCTCTGGATCCTGG 720

************************************************************

**BN4RT-R**

cT3 CCGCCCATCGCCCCGCCGCCCCAGGAGGCGGAGACGGATCCGTCCGTGCTCTGGCTCGCC 288

1BL CCGCCCGTGGCGCCGCCGCCGCAGGAGGCGGAGACGGATCCGTCCGTGCTCTGGCTCGCC 780

1AL CCGCCCATCGCCCCGCCGCCCCAGGAGGCGGAGACGGATCCGTCCGTGCTCTGGCTCGCC 627

1DL CCGCCCATCGCGCCGCCGCCGCAGGAGGCGGAGACGGATCCGTCCGTGCTCTGGCTCGCC 780

****** * ** ******** ***************************************

cT3 GACGCGCCGGCCGGGGACATGAAGGAGTCGCCGCCGTCCGTCGAGATCTCCGTCTCCAAG 348

1BL GACGCGCCGGCCGGGGACATGAAGGAGTCACCGCCGTCCGTCGAGATCTCCGTCTCCAAG 840

1AL GACGCGCCGGCCGGGGACATGAAGGAGTCGCCGCCGTCCGTCGAGATCTCCGTCTCCAAG 687

1DL GACGCGCCGGCCGGGGACATGAAGGAGTCGCCGCCGTCCGTCGAGATCTCCGTCTCCAAA 840

***************************** *****************************

cT3 CCGCCGCAGCCACAGCCGCCGCAGATCCATCAGTTCGAGAACGGGAGCACCAGCACGCTC 408

1BL CCGCCGCCGCCACAGCCGCCGCAGATCCATCACTTCGAGAACGGGAGCACCAGCACGCTC 900

1AL CCGCCGCAGCCACAGCCGCCGCAGATCCATCAGTTCGAGAACGGGAGCACCAGCACGCTC 747

1DL CCGCCGCCGCCACAACCGCCGCAGATCCATCACTTCGAGAACGGGAGCACCAGCACGCTC 900

******* ****** ***************** ***************************

cT3 ACGGAGAACCCCGGCCTCTCCGTGCACGCGCAGCAGCCTCCGCCGCAGCAGGCGGCCGCG 468

1BL ACGGAGAACCCCAGCCTGTCCGTGCACGCGCAGCAGCCTCCGCCACAGCAAGCGGCTGCG 960

1AL ACGGAGAACCCCGGCCTCTCCGTGCACGCGCAGCAGCCTCCGCCGCAGCAGGCGGCCGCG 807

1DL ACGGAGAACCCCAGTCTCTCCGTGCACGCGCAGCAGCCTCCGCCCCAGCAGGCGGCTGCG 960

************ * ** ************************** ***** ***** ***

cT3 GCGGCGCAGAGGCAGAACCAG------CACCAGCTCCAGCATCAGCACCAGCTCCAGCTC 522

1BL GCGGCGCAGAGGCAGAACCA------GCACCAGCTCCAGCATCAGCACCAGCTCCAACTC 1014

1AL GCGGCGCAGAGGCAGAACCA------GCACCAGCTCCAGCATCAGCACCAGCTCCAGCTC 861

1DL GCGGCGCAGAGGCAGAACCAGCACCAGCAGCAGCTCCAGCATCAGCACCAGCTCCAGCTC 1020

******************** ** ************************** ***

cT3 CAGCACCAGCACAATCAGGGTCCTTTCCGCCGGGAGCTCAATTTCTCAGATTTCGCGTCC 582

1BL CAGCACCAGCACAACCAGGGCCCTTTCCGCCGGGAGCTCAATTTCTCAGATTTCGCGTCC 1074

1AL CAGCACCAGCACAATCAGGGTCCTTTCCGCCGGGAGCTCAATTTCTCAGATTTCGCGTCC 921

1DL CAGCACCAGCACAACCAGGGTCCTTTCCGCCGGGAGCTCAATTTCTCAGATTTCGCGTCC 1080

************** ***** ***************************************

cT3 AACGCATCCGTCA----------------------------------------------- 595

1BL AACGCATCCGTCACGGTGACCCCGCCTTTCTTCAAGCCCGAGTCTGGTGAGATCCTAAAC 1134

1AL AACGCATCCGTCAAGGTGACCCCGCCTTTCTTCAAGCCTGAGTCTGGTGAGATCCTAAAC 981

1DL AACGCATCCGTCACGGTGACCCCGCCTTTCTTCAAGCCCGAGTCTGGTGAGATCCTAAAC 1140

*************

cT3 ------------------------------------------------------------ 595

1BL TTTGGCGCTGACAGCACCAGCCGGAGGAACCCTTCGCCGGCGCCCCCCGCCGCGACGGCC 1194

1AL TTTGGCGCTGACAGCACCAGCCGGAGGAACCCTTCGCCGGCACCCCCCGCCGCGACGGCC 1041

1DL TTTGGCGCTGACAGCACCAGCCGGAGGAACCCTTCGCCGGCGCCCCCCGCCGCGACGGCC 1200

cT3 ------------------------------------------------------------ 595

1BL AGCCTCACCACCGCGCCGGGGAGCCTCTTCTCCCAGCACACGGCGACTGTGACGGCCCCA 1254

1AL AGCCTCACCACCGCGCCGGGGAGCCTCTTCTCCCAGCACACGGCGACTGTGACGGCCCCA 1101

1DL AGCCTCACCACCGCGCCCGGGAGCCTATTCTCCCAGCACACGGCGACTGTGACGGCCCCG 1260

cT3 ------------------------------------------------------------ 595

1BL TCAAACGACGCCAAGAACAACCCGAAGCGGTCCATGGAGGCCACCTCCCGCGCGAGCAAC 1314

1AL TCAAACGACGCCAAGAACAACCCGAAGCGGTCCATGGAGGCCACCTCCCGCGCGAGCAAC 1161

1DL TCAAACGACGCCAAGAACAACCCGAAGCGGTCCATGGAGGCCACCTCCCGCGCGAGCAAC 1320

cT3 ------------------------------------------------------------ 595

1BL ACCAA-CACCACCAGACCGCCACAGCCAACGAGGGGATGCTGTCCTTCTCGTCGGCGCCG 1373

1AL ACCAACCACCACCAGACCGCGACAGCCAACGAGGGGATGCTGTCCTTCTCGTCGGCGCCG 1221

1DL ACCAACCACCACCAGAACGCCACAGCCAACGAGGGGATGCTGTCCTTCTCGTCGGCGCCG 1380

cT3 ------------------------------------------------------------ 595

1BL ACGACGCGGCCGTCCACCGGCACGGGCGCGCCAGCCAAGTCGGAGTCCGACCACTCCGAC 1433

1AL ACGACGCGGCCGTCCACCGGCACGGGCGCACCAGCCAAGTCGGAGTCCGACCACTCAGAC 1281

1DL ACGACGCGGCCGTCCACCGGCACGGGCGCGCCAGCCAAGTCGGAGTCCGACCACTCCGAC 1440

cT3 ------------------------------------------------------------ 595

1BL CTGGAGGCGTCGGTCCGCGAGGTGGAGAGCAGCCGCGTGGTGCCTCCGCCGGAGGAGAAG 1493

1AL CTGGAGGCGTCGGTCCGCGAGGTGGAGAGCAGCCGCGTGGTGCCTCCGCCGGAGGAGAAG 1341

1DL CTGGAGGCGTCGGTCCGCGAGGTGGAGAGCAGCCGCGTGGTGCCTCCGCCGGAGGAGAAG 1500

cT3 ------------------------------------------------------------ 595

1BL CGGCCGCGCAAGCGCGGGCGCAAGCCGGCGAACGGGCGCGAGGAGCCCCTGAACCACGTG 1553

1AL CGGCCGCGCAAGCGCGGGCGCAAGCCGGCGAACGGGCGCGAGGAGCCCCTGAACCACGTG 1401

1DL CGGCCGCGCAAGCGCGGGCGCAAGCCGGCGAACGGGCGCGAGGAGCCCCTGAACCACGTG 1560

cT3 -CGGCGGAGCGGCAGCGGCGAGAGAAGCTGAACCAGCGGTTCTACACGCTCCGCGCCGTG 654

1BL GAGGCGGAGCGGCAGCGGCGGGAGAAGCTGAACCAGCGGTTCTACGCGCTCCGCGCCGTG 1613

1AL GAGGCGGAGCGGCAGCGGCGAGAGAAGCTGAACCAGCGGTTCTACGCGCTCCGCGCCGTG 1461

1DL GAGGCGGAGCGGCAGCGGCGGGAGAAGCTGAACCAGCGGTTCTACGCCCTCCGCGCCGTG 1620

****************** ************************ * ************

cT3 GTGCCCAACGTGTCCAAGATGGACAAGGCCTCGCTGCTCGGCGACGCCATCTCCTACATC 714

1BL GTGCCCAACGTGTCCAAGATGGACAAGGCGTCGCTGCTCGGCGACGCCATCTCCTACATC 1673

1AL GTGCCCAACGTGTCCAAGATGGACAAGGCCTCGCTGCTCGGCGACGCCATCTCCTACATC 1521

1DL GTGCCCAACGTGTCCAAGATGGACAAGGCCTCGCTGCTGGGCGACGCCATCTCCTACATC 1680

***************************** ******** *********************

cT3 AACGAGCTTCGCGGCAAGATGACGGCGCTGGAGTCGGACAAGGAGACGCTCCATTCCCAA 774

1BL AACGAGCTCCGCGGCAAGATGACGGCGCTGGAGTCGGACAAGGAGACGCTCCATTCCCAA 1733

1AL AACGAGCTTCGCGGCAAGATGACGGCGCTGGAGTCGGACAAGGAGACGCTCCATTCCCAA 1581

1DL AACGAGCTCCGCGGCAAGATGACGGCGCTGGAGTCGGACAAGGAGACGCTCCACTCCCAA 1740

******** ******************************************** ******

cT3 ATCGAGGCGCTCAAGAAGGAGCGCGACGCCCGGCCGGCCGCGCCGTCGTCGGGGATGCAC 834

1BL ATCGAGGCGCTCAAGAAGGAGCGCGACGCCCGGCCGGCCGCGCCGTCGTCGGGGATGCAC 1793

1AL ATCGAGGCGCTCAAGAAGGAGCGCGACGCCCGGCCGGCCGCGCCGTCGTCGGGGATGCAC 1641

1DL ATCGAGGCGCTCAAGAAGGAGCGCGACGCCCGGCCGGCCGCGCCGTCGTCGGGGATGCAC 1800

************************************************************

cT3 GACAACGGGGCGCGGTGCCACGCGGTGGAGATCGAGGCCAAGATCCTGGGGCTGGAGGCG 894

1BL GACAACGGGGCGCGGTGCCACGCGGTGGAGATCGAGGCCAAGATCCTGGGGCTGGAGGCG 1853

1AL GACAACGGGGCGCGGTGCCACGCGGTGGAGATCGAGGCCAAGATCCTGGGGCTGGAGGCG 1701

1DL GACAACGGGGCGCGGTGCCACGCGGTCGAGATCGAGGCCAAGATCCTGGGGCTGGAGGCG 1860

************************** *********************************

cT3 ATGATCCGCGTGCAGTGCCACAAGCGCAACCACCCGGCGGCGAAGCTGATGACGGCGCTG 954

1BL ATGATCCGCGTGCAGTGCCACAAGCGCAACCACCCGGCGGCGAAGCTGATGACGGCGCTG 1913

1AL ATGATCCGCGTGCAGTGCCACAAGCGCAACCACCCGGCGGCGAAGCTGATGACGGCGCTG 1761

1DL ATGATCCGCGTGCAGTGCCACAAGCGCAACCACCCGGCGGCGAAGCTGATGACGGCGCTG 1920

************************************************************

cT3 CGGGAGCTGGACCTGGACGTGTACCACGCCAGCGTCTCGGTGGTGAAGGACATCATGATC 1014

1BL CGGGAGCTGGACCTGGACGTGTACCACGCCAGCGTCTCCGTGGTGAAGGACATCATGATC 1973

1AL CGGGAGCTGGACCTGGACGTGTACCACGCCAGCGTCTCGGTGGTGAAGGACATCATGATC 1821

1DL CGGGAGCTGGACCTGGACGTGTACCACGCCAGCGTGTCCGTGGTGAAGGACATCATGATC 1980

*********************************** ** *********************

cT3 CAGCAGGTGGCGGTGAAGATGGCCACCCGGGTCTACTCCCAGGACCAGCTCAACGCGGCG 1074

1BL CAGCAGGTGGCGGTGAAGATGGCCACCCGGGTGTACTCGCAGGACCAGCTCAACGCGGCG 2033

1AL CAGCAGGTGGCGGTGAAGATGGCCACCCGGGTCTACTCCCAGGACCAGCTCAACGCGGCG 1881

1DL CAGCAGGTGGCGGTGAAGATGGCCACCCGGGTCTACTCCCAGGACCAGCTCAACGCGGCG 2040

******************************** ***** *********************

cT3 CTCTACGGCCGCCTCGCCGAGCCGGGCACCGCGATGCAAATCCGCTAA 1122

1BL CTCTACGGCCGCCTCGCCGAGCCGGGCACCGCGATGCAAATCCGGTAA 2081

1AL CTCTACGGCCGCCTCGCCGAGCCGGGCACCGCGATGCAAATCCGGTAA 1929

1DL CTCTACGGCCGCCTCGCCGAGCCGGGCACCGCGATGCAAATCCGGTAA 2088

******************************************** ***

**File S2**: ***TaMYC4* homeologs protein sequence for domain prediction.**

>1AL-Protein (693 aa); 52-228: bHLH domain, 753-774: HLH DNA binding domain

MNLWTDDNASMMEAFMAXXXXXXXXXXXXXXXXXXXXXXXXXXXXXXXXXXRLQAIIEGSRETWTYAIFWQSSTDAGASLLGWGDGYYKGCDDADKRRQQPTPASAAEQEHRKRVLRELNSLIAGGGAAAPDEAVEEEVTDTEWFFLVSMTQSFPNGMGLPGQALFAGQPTWIATGLASAPCERARQAYTFGLRTMVCIPLGTGVLELGATEVIFQTNDSLGRIRSLFNLNGGGGGSGSWPPIAPPPQEAETDPSVLWLADAPAGDMKESPPSVEISVSKPPQPQPPQIHQFENGSTSTLTENPGLSVHAQQPPPQQAAAAAQRQNQHQLQHQHQLQLQHQHNQGPFRRELNFSDFASNASVKVTPPFFKPESGEILNFGADSTSRRNPSPAPPAATASLTTAPGSLFSQHTATVTAPSNDAKNNPKRSMEATSRASNTNHHQTATANEGMLSFSSAPTTRPSTGTGAPAKSESDHSDLEASVREVESSRVVPPPEEKRPRKRGRKPANGREEPLNHVEAERQRREKLNQRFYALRAVVPNVSKMDKASLLGDAISYINELRGKMTALESDKETLHSQIEALKKERDARPAAPSSGMHDNGARCHAVEIEAKILGLEAMIRVQCHKRNHPAAKLMTALRELDLDVYHASVSVVKDIMIQQVAVKMATRVYSQDQLNAALYGRLAEPGTAMQIR

>1BL-Protein (673 aa); 49- 228: bHLH MYC TF domain, 514-561: HLH DNA binding domain, 556-589: bZIP domain, 683: mutation site

MNLWTDDNASMMEAFMASADMPAFPWGAAATPPPPAAVPQQPAFNQDTLQQRLQAIIEGSRETWTYAIFWQSSTDAGASLLGWGDGYYKGCDDADKRRQQPTPASAAEQEHRKRVLRELNSLIAGGGAAAPDEAVEEEVTDTEWFFLVSMTQSFPNGMGLPGQALFAGQPTWIATGLASAPCERARQAYTFGLRTMVCIPLGTGVLELGATEVIFQTNDSLGRIRSLFNLNGGGGGSGSWPPVAPPPQEAETDPSVLWLADAPAGDMKESPPSVEISVSKPPPPQPPQIHHFENGSTSTLTENPSLSVHAQQPPPQQAAAAAQRQNQHQLQHQHQLQLQHQHNQGPFRRELNFSDFASNASVTVTPPFFKPESGEILNFGADSTSRRNPSPAPPAATASLTTAPGSLFSQHTATVTAPSNDAKNNPKRSMEATSRASNTNHHQTATANEGMLSFSSAPTTRPSTGTGAPAKSESDHSDLEASVREVESSRVVPPPEEKRPRKRGRKPANGREEPLNHVEAERQRREKLNQRFYALRAVVPNVSKMDKASLLGDAISYINELRGKMTALESDKETLHSQIEALKKERDARPAAPSSGMHDNGARCHAVEIEAKILGLEAMIRVQCHKRNHPAAKLMTALRELDLDVYHASVSVVKDIMIQQVAVKMATRVYSQDQLNAALYGRLAEPGTAMQIR

>1DL-Protein (695 aa); 49- 228: bHLH domain, 516-564: HLH DNA binding domain

MNLWTDDNASMMEAFMASADMPAFPWGAAATPPPPAAVPQQPAFNQDTLQQRLQAIIEGSRETWTYAIFWQSSTDAGASLLGWGDGYYKGCDDADKRRQQPTPASAAEQEHRKRVLRELNSLIAGGGAAAPDEAVEEEVTDTEWFFLVSMTQSFPNGMGLPGQALFAGQATWIATGLASAPCERARQAYTFGLRTMVCIPLGTGVLELGATEVIFQTNDSLGRIRSLFNLNGGGGGSGSWPPIAPPPQEAETDPSVLWLADAPAGDMKESPPSVEISVSKPPPPQPPQIHHFENGSTSTLTENPSLSVHAQQPPPQQAAAAAQRQNQHQQQLQHQHQLQLQHQHNQGPFRRELNFSDFASNASVTVTPPFFKPESGEILNFGADSTSRRNPSPAPPAATASLTTAPGSLFSQHTATVTAPSNDAKNNPKRSMEATSRASNTNHHQNATANEGMLSFSSAPTTRPSTGTGAPAKSESDHSDLEASVREVESSRVVPPPEEKRPRKRGRKPANGREEPLNHVEAERQRREKLNQRFYALRAVVPNVSKMDKASLLGDAISYINELRGKMTALESDKETLHSQIEALKKERDARPAAPSSGMHDNGARCHAVEIEAKILGLEAMIRVQCHKRNHPAAKLMTALRELDLDVYHASVSVVKDIMIQQVAVKMATRVYSQDQLNAALYGRLAEPGTAMQIR
